# An evaluation of the Acromegaly Treatment Satisfaction Questionnaire (Acro-TSQ) in adult patients with acromegaly, including correlations with other patient-reported outcome measures: data from two large multicenter international studies

**DOI:** 10.1007/s11102-020-01038-y

**Published:** 2020-03-27

**Authors:** Maria Fleseriu, Leon Fogelfeld, Murray B. Gordon, Jill Sisco, Ross D. Crosby, William H. Ludlam, Asi Haviv, Susan D. Mathias

**Affiliations:** 1 Departments of Medicine and Neurological Surgery and Northwest Pituitary Center, Oregon Health and Science University, 3303 SW Bond Ave, CH8N, Portland, OR 97239 USA; 2 John H. Stroger Jr. Hospital of Cook County, Rush University Medical Center, Chicago, IL USA; 3 Allegheny Neuroendocrinology Center, Allegheny General Hospital, Pittsburgh, PA USA; 4 Acromegaly Community, Grove, OK USA; 5 Health Outcomes Solutions, Winter Park, FL USA; 6 Neuropsychiatric Research Institute, Fargo, ND USA; 7 University of North Dakota School of Medicine and Health Sciences, Fargo, ND USA; 8 Chiasma, Inc., Waltham, MA USA

**Keywords:** Acromegaly, Patient reported outcomes, Quality of life, Acro-TSQ, Validation, Measurement properties, Questionnaire

## Abstract

**Purpose:**

The Acromegaly Treatment Satisfaction Questionnaire (Acro-TSQ) is a new patient-reported outcome (PRO) measure for patients with acromegaly receiving injectable somatostatin analogs (SSAs) to assess clinical symptoms and adverse drug reaction interference, treatment satisfaction, and convenience. We evaluated its scale structure, reliability, validity, responsiveness, and what constitutes clinically meaningful change.

**Methods:**

Data from two longitudinal studies (N = 79 and 82) of patients receiving a stable injectable SSA dose for ≥ 6 months who completed the Acro-TSQ and other collateral measures (e.g., AcroQoL, AIS, WPAI:SHP, EQ-5D-5L) were analyzed.

**Results:**

The first study demonstrated internal consistency of the Acro-TSQ. However, several items had high ceiling effects, responsiveness could not be established, and the minimally important difference (MID) was not estimable. In the second study, factor analysis revealed six scales: Symptom Interference, Treatment Convenience, Injection Site Interference, GI Interference, Treatment Satisfaction, and Emotional Reaction. Internal consistency and test–retest reliability were confirmed; most scales demonstrated significant differences in mean scores by disease severity. Correlations between Acro-TSQ scales and other collateral measures exceeded 0.30 in absolute value, confirming convergent validity. Responsiveness in Acro-TSQ scale scores reflected improved disease control. The MID was estimated for Symptom Interference (10–12 points), Treatment Convenience (9–11) and GI Interference (8–10).

**Conclusions:**

The Acro-TSQ is a brief, yet comprehensive tool to monitor important outcomes associated with injectable acromegaly SSA treatments. Its content reflects both disease and treatment burden as well as patient satisfaction, and its relevant for use in clinical studies.

**Electronic supplementary material:**

The online version of this article (10.1007/s11102-020-01038-y) contains supplementary material, which is available to authorized users.

## Introduction

Acromegaly is a rare hormonal disorder where excess growth hormone (GH) and insulin-like growth factor 1 (IGF-1) are produced; it is most often caused by a benign tumor of the pituitary gland [1–3]. Patients can experience changes to their appearance and enlargement of hands and feet, as well as other symptoms and comorbidities [4]. First-line medical treatment is commonly somatostatin analogs (SSAs) [5–7] administered as either intramuscular (octreotide LAR) or deep subcutaneous injections (lanreotide depot); however, common side effects can include injection site reactions and gastrointestinal (GI) symptoms [8, 9]. Despite achieving biomedical control as defined by normal IGF-1 and GH levels, acromegaly symptoms may persist [10]. Acromegaly symptoms and treatment-related side effects can negatively impact patients’ health-related quality of life (HRQoL) [10–13].

A new patient-reported outcome (PRO) measure, the Acromegaly Treatment Satisfaction Questionnaire (Acro-TSQ) [14], has been developed specifically for use with acromegaly patients receiving injectable SSA treatment to assess symptom and GI side effect interference, treatment satisfaction, treatment bother, and treatment convenience. The Acro-TSQ was developed based on qualitative research with patients with acromegaly and input from endocrinologists, and found to be comprehensive, clear, and relevant for this population [15, 16].

Here we present the results of the examination of the measurement properties of the Acro-TSQ, including its factor structure, reliability, and validity; these results reflect the findings from two separate studies (described below). We also present the evaluation of what constitutes a clinically meaningful change.

## Methods

The evaluation and confirmation of the measurement properties of the Acro-TSQ involved two studies; results of the first study prompted modifications to the Acro-TSQ, which was then re-evaluated during the second study.

### First study

Fourteen sites in the United States, the United Kingdom, and the Netherlands, as well as the Acromegaly Community (a US-based non-profit organization that offers support for those affected by acromegaly, found at https://www.acromegalycommunity.org), identified, enrolled, and consented individuals with acromegaly to participate in the study. Human subjects’ research approval was provided by an independent, scientific review committee, The Copernicus Group, Cary; all academic clinical sites obtained their own local institutional review board (IRB) approval. To be eligible, patients were required to be aged 18 to 75 years, have documented evidence of a GH-secreting pituitary tumor that is abnormally responsive to glucose or abnormal IGF-1 values as a diagnosis of acromegaly, be currently receiving injectable SSA (octreotide LAR or lanreotide depot) therapy at a stable dose for at least 6 months, be able to speak, read, and write English and consent to participation, and be willing to complete the Acro-TSQ and collateral PRO questionnaires up to three times during a 3-month period of time. Patients were excluded if they had participated in the qualitative research used in the development stage of the Acro-TSQ or if there was evidence of a medical condition or treatment for a condition that results in a cognitive or other (visual, hearing) impairment that would interfere with participating in the study (based on the screener’s opinion).

Sites completed a Case Report Form (CRF) at baseline and upon completion of the study for each patient enrolled. The baseline CRF included items assessing the date of diagnosis, level of disease control, lab values, and prior and current treatments for acromegaly. The follow-up CRF asked about updates within the past 3 months in terms of any recent lab tests, changes in acromegaly treatment, and also contained a clinician global impression of change (CGI-C) item related to acromegaly symptoms. This study was observational and did not require any change in patients’ medical treatment during the assessment period.

Subjects were asked to complete the Acro-TSQ, as well as collateral questionnaires (including the Treatment Satisfaction Questionnaire for Medication (TSQM) [17], the Acro-QoL [18], and a Patient Global Assessment (PGA) at Baseline, Retest (7–10 days later for a subset of patients), and Month 3. Patients also completed a Patient Global Impression of Change (PGI-C) rating at Retest and Month 3, which asks whether patients experienced a change in severity of their acromegaly symptoms since baseline. Preliminary results from this study have been presented previously in poster form [15, 16].

### Second study

Some results from the first study suggested that modifications to Acro-TSQ items were necessary. Specifically, test–retest reliability was below the desired threshold and some item-level ceiling effects were identified. Additionally, it was not possible to estimate what constitutes a minimally important difference (MID) as no changes in medical treatment or disease activity occurred during the assessment period. Items related to emotions and injection site reactions were modified, and the measurement properties of the updated version of the Acro-TSQ were re-evaluated in the second study to establish its scale structure, validity, reliability, and to estimate the MIDs.

Data for these analyses were obtained from the Phase III MPOWERED study [19] that was performed to evaluate the efficacy and safety of acromegaly patients treated with octreotide capsules and to examine PROs.

In the initial screening and run-in phases of this study, patients controlled with injectable SSAs switched to oral octreotide for a period of 26 weeks. Data collected during the screening and run-in phases (the beginning of the run-in phase is referred to as ‘baseline’; the end as ‘week 26’) were used. Longitudinal analyses utilizing change scores were performed for all patients for whom the updated Acro-TSQ versions were available at both screening and week 26. More details on the MPOWERED study design were presented in poster form [19] and are also available elsewhere (clinical trial NCT 02685709).

Several PRO assessments were administered as part of this study, including the updated version of the Acro-TSQ, the Acromegaly Index of Severity (AIS), the Work Productivity and Activity Impairment Questionnaire Specific Health Problem V2.0 (WPAI:SHP) [20], and the 5-level EQ-5D version (EQ-5D-5L) [21]. The Acro-TSQ includes 24 questions which cover the following concepts: symptoms/symptom control, interference with symptoms, GI side effects, interference with GI side effects and injection site reactions and their impact, emotional impacts of treatment, convenience/ease of treatment, and overall satisfaction. The Acro-TSQ was self-administered by patients at screening, baseline, and week 26 (end of run-in phase). Not all questions were asked at week 26 because all patients received an oral formulation during the run-in phase, therefore some questions referring specifically to injectable treatments were not relevant. Details regarding the AIS, WPAI:SHP, and EQ-5D-5L can be found in the supplementary material.

### Statistical methods

For both studies, frequencies and percentages or means, standard deviations (SDs), and ranges are reported on demographic and clinical characteristics. Descriptive information on collateral measures are reported at baseline and month 3 (first study) or at screening, baseline, and week 26 (second study). Individual item responses were also examined at baseline and month 3 (first study) or at screening, baseline, and week 26 (second study); this included the frequency/percentage of each possible response, mean and SD where applicable, and the frequency and percentage of floor (most symptomatic) and ceiling (least symptomatic) responses.

### Exploratory factor analysis (EFA)

Exploratory factor analysis [22] was employed to reveal the underlying structure of the Acro-TSQ to produce scale scores. For both studies, EFA was performed using Acro-TSQ items at either baseline (first study) or screening (second study) using maximum likelihood extraction and Promax non-orthogonal rotation.

### Acro-TSQ scale analysis

For each Acro-TSQ scale identified by EFA, scoring algorithms were developed to produce a scale score ranging from 0 to 100, with 0 representing the lowest satisfaction/highest interference and 100 representing the highest satisfaction/lowest interference. Scale scores were only calculated when at least 50% of the items within that scale had valid responses. Descriptive information on Acro-TSQ scale scores at baseline and month 3 (first study) or at screening, baseline, and week 26 (second study) were calculated, including means, medians, SDs, ranges, and frequency and percentage of floor (i.e., most symptomatic) and ceiling (i.e., least symptomatic) responses.

### Reliability

Internal consistency reliability is a measure of how closely items that aim to measure the same general construct produce similar results. This was evaluated for each Acro-TSQ scale at baseline and month 3 (first study) or at screening, baseline, and week 26 (second study) using Cronbach’s alpha coefficients [23]. Test–retest reliability (the extent to which the Acro-TSQ produces the same results over repeated applications in an unchanged population) for each scale was evaluated by calculating the intra-class correlation coefficient (ICC) [24] between either the baseline and retest (first study) or between the screening and baseline (second study) assessments. For the first study, every other subject enrolled at each site was asked to complete the retest assessment. Only those individuals whose PGA rating at retest indicated that their acromegaly symptoms had not changed since baseline were included in this analysis. For the second study, test–retest data were restricted to those patients whose baseline EQ-VAS score was within ± 5% of their score from the screening assessment.

### Convergent validity

To determine whether Acro-TSQ scales correlate with other tools that measure similar concepts, convergent validity was assessed by examining the Pearson correlations of the Acro-TSQ scales with the TSQM and AcroQoL at baseline and month 3 for the first study, and the AIS, WPAI:SHP, EQ-5D-5L, and IGF-1 at screening, baseline, and week 26 for the second study. A minimum correlation of 0.30 (a “medium” effect size as defined by Cohen, 1988 [25]) between conceptually similar scales was required for evidence of convergent validity.

### Known groups validity

Known groups validity measures the ability of the Acro-TSQ to discriminate between groups known to be clinically different. For the first study, known-groups validity was evaluated by categorizing patients into groups according to different clinical parameters at baseline and comparing these groups on Acro-TSQ scores. These clinical parameters included baseline PGA severity ratings (absent, mild, moderate, severe, very severe), assessment of control of acromegaly at month 3 (well controlled, partially controlled, not controlled), change in acromegaly treatment regimen during the study, and time since initiation of SSA treatment. The categorization of patients was determined based upon the observed distributions to achieve relative balance in sample size across categories. Comparisons between groups on baseline Acro-TSQ scale scores were made using analysis of variance.

Known groups validity in the second study was evaluated as described for the first study, except that it used the AIS Overall Score and IGF-1 values. AIS Overall Scores at screening were divided into three categories (Low = 0–3, Medium = 4–7, High ≥ 7), which resulted in relatively comparably sized groups. The cutoffs for IGF-1 were based upon clinical treatment guidelines [7, 26] with the following cutoffs: ≤ 1.0 upper limits of normal (ULN) and > 1.0 ULN.

### Responsiveness

To assess the ability of the Acro-TSQ to detect changes over time, several measures of responsiveness were used, including the standardized effect size (SES) [25], the standardized response mean (SRM) [27], and the responsiveness statistic (RS) [28].

### MID

The MID represents the minimum change that can be considered to be clinically relevant. The determination of the MID incorporated both anchor- and distribution-based methods [29]. Anchor-based methods link scales to patient- or provider-reported change ratings, while distribution-based methods examine the statistical distribution of measure scores.

A final MID range was established that integrates estimates from both distribution- and anchor-based methods.

More detailed descriptions of the methods employed for EFA, known-groups validity, responsiveness, and MID estimation can be found in the supplementary material.

### Sample size

To a large extent, the sample size is pragmatic because acromegaly is a rare condition. Specifically, for the first study the protocol stipulated that at least 50 subjects would be enrolled. This sample size is considered to be the minimum needed to conduct EFA and will provide sufficient power (0.80) to detect a correlation of 0.36 between Acro-TSQ scales and collateral measures used to evaluate convergent validity. For the second study, we used all available data that met the entry criteria for inclusion.

## Results

### First study

The first study collected prospective data from 79 individuals with acromegaly. EFA revealed five factors (Acromegaly Symptoms, Treatment-Related GI Side Effects, Treatment Satisfaction, Treatment Administration Bother, and Treatment Convenience). Good internal consistency reliability was found for all scales; however, test–retest reliability was below the established standard of 0.70 for two scales (Treatment-related GI Side Effects [0.61] and Treatment Convenience [0.61]), which suggested that these scales may have some instability over the 7- to 10-day test–retest period. Multiple items related to injection site reaction interference and emotional items demonstrated high ceiling effects, or the percent of responses at the highest response option. For example, when asked how much injection site reactions interfered with the ability to do daily activities, the highest response option possible was “Not at all,” followed by several options describing some level of interference ranging from “A little bit” to “Very much.” High ceiling effects for these items (range at baseline: 49% to 73%; range at follow-up: 44% to 71%), indicates that most patients did not experience them.

Convergent and known groups validity was demonstrated for all scales. However, the responsiveness of the Acro-TSQ scales could not be established because individuals enrolled in this study were relatively stable over time: 60% of patient ratings (PGI-C) and 87% of clinical ratings (CGI-C) indicated no change in acromegaly symptoms. In addition, no suitable anchors were identified for estimating an anchor-based MID because none of the Acro-TSQ scales demonstrated a correlation with patient- or provider-reported impressions of change that reached the minimum threshold of 0.30. Therefore, the data did not allow for an estimate of the amount of change in Acro-TSQ scales that corresponded to what either the patient or provider considered to be meaningful change.

**Fig. 1:**
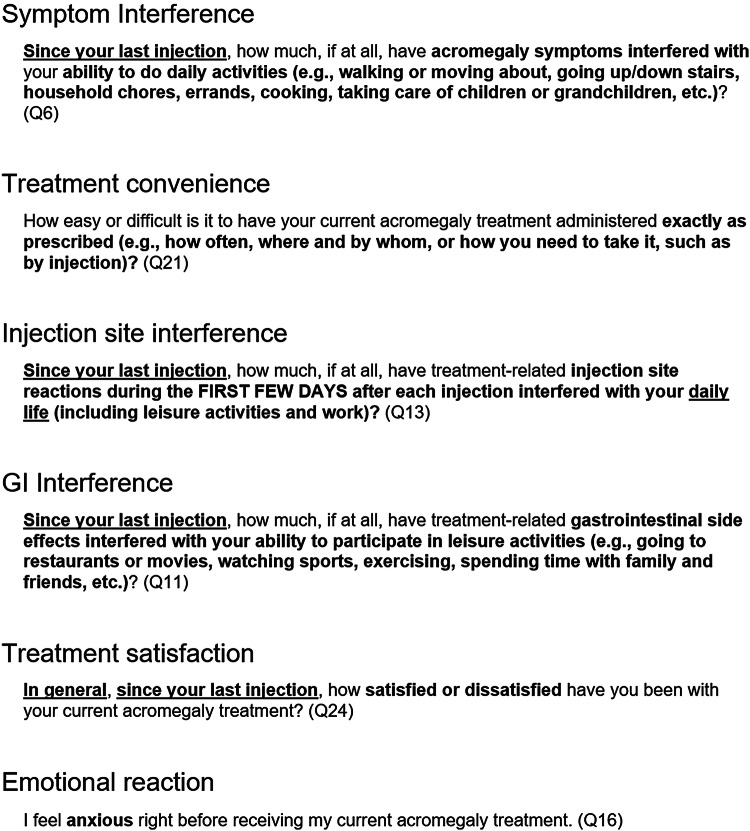
Sample items from the Acro-TSQ by scale (with the associated item number in parentheses)

Based on these results several Acro-TSQ items were modified. For example, while patients in the first study were asked to rate the severity of emotions (sad, anxious, or frustrated/angry) about receiving acromegaly treatment on a scale ranging from 0 to 10, the updated version asked how much they experienced feelings of sadness, anxiety, or frustration about receiving treatment, with response options of “Not at all,” “A little bit,” “Somewhat,” “Quite a bit,” or “Very much.” The updated version of the Acro-TSQ was incorporated into the Phase 3, randomized, open-label, active controlled, multi-center study (MPOWERED; OOC-ACM-302) [19]. Figure 1 contains sample items from each scale of the Acro-TSQ.

### Second study

The second study included data from 82 patients, including 54 females (66%) and 28 males (34%) with an average age of 53 (SD = 11) years. The majority of participants (94%) resided outside the US. The average time since acromegaly diagnosis was 11 years (SD = 8), and the mean IGF-1 ULN at baseline was 0.86 (SD = 0.26), with 65% of patients having an IGF-1 ≤ 1.0 ULN. Mean GH at baseline was 0.85 (SD = 0.62); 61% had GH < 1.0 ng/mL, 37% had GH between 1 and 2.5 ng/mL, and 1% (one patient) had GH > 2.5 ng/mL.

#### Exploratory factor analysis (EFA)

The EFA yielded six factors that accounted for 76% of the criterion variance. The six factors were: Symptom Interference, Treatment Convenience (a blend of items from the Treatment Bother and Treatment Convenience factors in the first study), Injection Site Interference, GI Interference, Treatment Satisfaction, and Emotional Reaction.

Analyses of Acro-TSQ scales revealed high rates of ceiling effects (i.e., least symptomatic) for Symptom Interference (screening, baseline, and week 26), Injection Site Interference (screening and baseline), GI Interference (screening, baseline, and week 26), and Emotional Reaction (screening, baseline, and week 26) (range: 27% to 53%, Table 1). The first three of these were primarily due to skip out questions, questions that are only relevant for those who answer a certain way to previous questions and therefore are not completed by all respondents.

**Table 1:** Acro-TSQ score descriptive summary, second study

Acro-TSQ scale	Statistic	Screening N = 82	Baseline N = 81	Week 26 N = 77
Symptom interference	Mean	72	78	78
	Median	75	81	94
	SD	25	24	27
	Range	19–100	19–100	0–100
	Floor (n, %)	0 (0%)	0 (0%)	1 (1%)
	Ceiling (n, %)	26 (32%)	35 (43%)	38 (49%)
Treatment convenience	Mean	67	68	70
	Median	67	71	71
	SD	21	21	18
	Range	4–100	17–100	17–100
	Floor (n, %)	0 (0%)	0 (0%)	0 (0%)
	Ceiling (n, %)	4 (5%)	5 (6%)	1 (1%)
Injection site interference^a^	Mean	84	85	
	Median	88	88	
	SD	20	18	
	Range	13–100	38–100	
	Floor (n, %)	0 (0%)	0 (0%)	
	Ceiling (n, %)	39 (48%)	40 (49%)	
GI interference	Mean	80	84	83
	Median	92	92	100
	SD	24	21	23
	Range	0–100	17–100	25–100
	Floor (n, %)	1 (1%)	0 (0%)	0 (0%)
	Ceiling (n, %)	37 (45%)	39 (48%)	41 (53%)
Treatment satisfaction	Mean	61	61	58
	Median	61	61	61
	SD	20	19	25
	Range	0–100	14–100	0–100
	Floor (n, %)	1 (1%)	0 (0%)	3 (4%)
	Ceiling (n, %)	2 (2%)	3 (4%)	3 (4%)
Emotional reaction	Mean	80	82	84
	Median	83	83	92
	SD	22	20	21
	Range	0–100	0–100	25–100
	Floor (n, %)	2 (2%)	2 (3%)	0 (0%)
	Ceiling (n, %)	23 (28%)	22 (27%)	33 (43%)

Floor is most symptomatic; Ceiling Least Symptomatic. Lower scores indicate greater interference/lower satisfaction; higher score indicate less interference/higher satisfaction

*GI* gastrointestinal, *SD* standard deviation

^a^Injection site interference scale was not administered at week 26

#### Reliability

Reliability analyses demonstrated that all Acro-TSQ scales had ICCs > 0.70 at all assessments (range 0.72 to 0.97), and test–retest reliability was > 0.70 for all Acro-TSQ scales (range 0.78 to 0.91) except Emotional Reaction, where the coefficient was 0.69. Not all questions were asked at week 26, so the ICC could not be calculated for the Injection Site Interference scale.

#### Convergent validity

When assessing convergent validity, the Symptom Interference scale consistently showed Pearson correlations > 0.30 in absolute value with the AIS Overall Score, WPAI:SHP Activity Impairment, and all EQ-5D-5L scores at screening, baseline (Table 2), and week 26. Treatment Convenience and Injection Site Interference scores also showed consistent correlations > 0.30 with WPAI:SHP Activity Impairment and all EQ-5D-5L scores across assessment points, but the correlations were lower with AIS scores. GI Interference and Treatment Satisfaction generally exhibited correlations > 0.30 with AIS Overall Scores, WPAI:SHP Activity Impairment, and most EQ-5D-5L scores at screening, baseline, and week 26. The only Acro-TSQ scale for which the correlation with IGF-1 ULN exceeded 0.30 was Treatment Satisfaction (which encompasses items about the extent to which treatment improves symptoms, the length of time the treatment lasted, and overall satisfaction with treatment), at both screening and week 26. Finally, Emotional Reaction consistently showed correlations > 0.30 with Anxiety/Depression from the EQ-5D-5L across all three assessment points.

**Table 2:** Convergent validity of the Acro-TSQ, first and second study

Measure	Scale	Acromegaly symptoms	Treatment related GI side effects	Treatment satisfaction	Treatment administration bother	Treatment Convenience
Acro-TSQ scales, first study						
TSQM	Effectiveness	0.29*	0.15	0.83**	0.31**	0.32**
	Side effects	0.15	0.36*	0.28*	0.44**	0.15
	Convenience	− 0.04	0.04	0.54**	0.60**	0.46**
	Overall satisfaction	0.21	0.31*	0.82**	0.27*	0.18
AcroQoL	Physical	0.58**	0.45**	0.41**	0.08	0.08
	Psychological	0.32*	0.46**	0.40**	0.02	0.001
	Physical appearance	0.30*	0.40**	0.45**	0.09	− 0.001
	Personal relationships	0.28*	0.44**	0.30**	− 0.06	0.001
	Global	0.46**	0.47**	0.43**	0.05	0.03

Measure	Scale	Symptom interference	Treatment convenience	Injection site interference	GI interference	Treatment satisfaction	Emotional reaction
Acro-TSQ scales, second study							
AIS	Overall	− 0.54**	− 0.22*	− 0.39**	− 0.25*	− 0.44**	− 0.18
WPAI:SHP	Absenteeism	− 0.09	− 0.34*	**− 0.32**	− 0.36*	− 0.25	− 0.41*
	Presenteeism	− 0.36*	− 0.39*	− 0.34*	− 0.56**	− 0.28	− 0.51**
	Work Productivity Loss	− 0.41*	**− 0.31**	− 0.26	− 0.46**	− 0.23	− 0.42**
	Activity Impairment	− 0.56**	− 0.48**	− 0.40**	− 0.42**	− 0.54**	− 0.37**
EQ-5D-5L	Mobility	− 0.50**	− 0.40**	− 0.38**	− 0.33**	− 0.42**	− 0.30**
	Self-care	− 0.39**	− 0.40**	− 0.45**	− 0.47**	− 0.39**	− 0.36**
	Usual activities	− 0.58**	− 0.40**	− 0.42**	− 0.39**	− 0.45**	− 0.35**
	Pain/discomfort	− 0.56**	− 0.39**	− 0.37**	− 0.48**	− 0.48**	− 0.27*
	Anxiety/depression	− 0.35**	− 0.38**	− 0.14	− 0.28*	− 0.37**	− 0.37**
	EQ-VAS	0.49**	0.31**	0.36**	0.24*	0.37**	0.30**
IGF-1		− 0.27*	− 0.05	− 0.07	− 0.05	− 0.25*	− 0.07

Cell values are Pearson correlations at baseline of each study

*p < 0.05; **p < 0.01

Negative correlations represent situations where high scores on the Acro-TSQ scale correspond to lower scores on the collateral measure

Bold values indicate correlations > 0.30 in absolute value

#### Known-groups validity

When categorizing patients based on AIS Overall Scores (Low = 0–3, Medium = 4–7, High ≥ 7) to assess known groups validity, significant differences between groups were found on all Acro-TSQ scales, with the exception of Treatment Convenience (p = 0.071). Large effects were found on three of the five remaining scales (eta-squared of 0.18 for Symptom Interference, 0.14 for Injection Site, 0.15 for GI Interference, Table 3). When categorizing patients based on IGF-1 ULN, the only significant difference between groups that was found was on Treatment Satisfaction (p = 0.016).

**Table 3:** Known-groups validity of the Acro-TSQ, second study

	Grouped by AIS overall score				
Acro-TSQ scale	AIS overall score group			Significance	Eta-Squared
	Low 0–3	Medium 4–7	High > 7		
Symptom interference (mean, SD, N)	86 (21) N = 29	66 (23) N = 30	61 (26) N = 23	F = 8.9; df = 2, 79; p < .001	0.18
Treatment convenience (mean, SD, N)	72 (18) N = 29	69 (22) N = 30	59 (22) N = 23	F = 2.7; df = 2, 79; p = .071	0.07
Injection site interference (mean, SD, N)	89 (15) N = 29	88 (15) N = 30	72 (27) N = 23	F = 6.3; df = 2, 79; p = .003	0.14
GI interference (mean, SD, N)	88 (18) N = 29	84 (21) N = 30	66 (28) N = 23	F = 6.7; df = 2, 79; p = .002	0.15
Treatment satisfaction (mean, SD, N)	70 (16) N = 29	60 (13) N = 30	52 (26) N = 23	F = 6.2; df = 2, 79; p = .003	0.14
Emotional reaction (mean, SD, N)	87 (17) N = 29	81 (19) N = 30	69 (29) N = 23	F = 4.5; df = 2, 79; p = .014	0.10

*AIS* Acromegaly Index of Severity, *Df* degree of freedom, *GI* gastrointestinal, *SD* standard deviation

#### Responsiveness

Analyses of responsiveness demonstrated that the Improved group, as defined by AIS Overall Score (Table 4) and IGF-1 changes (data not shown), showed larger improvements (i.e., positive coefficients) than the Unchanged and/or Worse group for every scale.

**Table 4:** Responsiveness of Acro-TSQ scales by changes in AIS Overall Score from screening to week 26, second study

Acro-TSQ scale	AIS change^a^	Standardized effect size	Standardized response mean	Responsiveness statistic
Symptom interference	Worse	0.03	0.03	0.07
	Unchanged	0.23	0.30	**0.59**
	Improved	0.35	0.45	**0.67**
	Overall	0.20	0.23	0.47
Treatment convenience	Worse	0.03	0.02	0.03
	Unchanged	− 0.32	− 0.32	− 0.33
	Improved	0.26	0.24	0.32
	Overall	0.06	0.05	0.07
GI interference	Worse	− 0.40	− 0.25	− 0.43
	Unchanged	0.22	0.28	0.32
	Improved	0.29	0.25	0.38
	Overall	0.09	0.07	0.11
Treatment satisfaction	Worse	− **0.72**	− 0.49	− **0.74**
	Unchanged	− 0.40	− 0.30	− 0.36
	Improved	0.18	0.18	0.17
	Overall	− 0.25	− 0.19	− 0.24
Emotional reaction	Worse	− 0.16	− 0.12	− 0.16
	Unchanged	− 0.19	− 0.13	− 0.08
	Improved	**0.50**	0.40	0.39
	Overall	0.13	0.10	0.10

Negative coefficients indicate worsening, while positive coefficients indicate improvement

*AIS* Acromegaly Index of Severity, *GI *gastrointestinal

^a^Sample size in each case is based on the actual number out of the overall (N = 77) whose AIS Overall Score at Week 26 was worse (n = 24), unchanged (n = 19), or improved (n = 34) from baseline

0.2 = “small” effect; 0.5 = “medium” effect; 0.8 = “large” effect; bold cells reflect medium or large effects

#### MID estimation

No preliminary MID estimates could be calculated in the first study because patients experienced little clinical change during the assessment period. In the second study, correlations for the EQ-VAS exceeded 0.30 for all Acro-TSQ scales except Emotional Reaction (0.25) and Injection Site Interference (which was not assessed at Week 26), confirming support for the EQ-VAS as a suitable anchor for Symptom Interference, Treatment Convenience, GI Interference, and Treatment Satisfaction. In contrast, neither IGF-1 ULN (for any scale) nor AIS Overall Score (for Symptom Interference) were supported for use as anchors. In the Unchanged Group (as defined by no change in EQ-VAS, N = 21), Acro-TSQ scores improved on the Treatment Convenience, GI Interference, and Treatment Satisfaction scales and worsened on the Symptom Interference score. In the Much Improved group (> 10 point increase in EQ-VAS, N = 11), scores increased in the Symptom Interference, Treatment Convenience, GI Interference, and Treatment Satisfaction scales.

The regression coefficients for the intercept (which indicate the amount of change expected in Acro-TSQ scale scores given no change in EQ-VAS) ranged from a decrease of 7 points for Symptom Interference to an increase of 27 points for Treatment Satisfaction. The regression coefficients for EQ-VAS change indicate the amount of change in Acro-TSQ scale scores for each 1-point change in EQ-VAS score. These values range from 0.48 for Symptom Interference to 0.84 for Treatment Satisfaction (data not shown).

Several distribution-based estimates for Acro-TSQ scales were obtained. SEM estimates ranged from 7 (Treatment Satisfaction) to 12 (Emotional Reaction), and 0.5 Cohen’s d estimates ranged from 10 (Treatment Satisfaction) to 13 (Symptom Interference).

After combining anchor- and distribution-based methods, MID estimates were established as ranges of scores (Table 5). The MID range proposed is 10–12 points for Symptom Interference, is 9–11 points for Treatment Convenience, and is 8–10 points for GI Interference. Given the large regression intercept for Treatment Satisfaction (27) along with the large observed change in the EQ-VAS Much Improved group (28), no estimate is provided for this scale. Further work is needed to establish the MID for this scale, along with the Injection Site Interference and the Emotional Reaction scales.

**Table 5:** Integration of anchor-based and distribution-based estimates for Acro-TSQ scales, second study

	Acro-TSQ scale					
Property	Symptom interference	Treatment convenience	Injection site interference	GI interference	Treatment satisfaction	Emotional reaction
Minimal detectable change	9–13	8–11	10	7–12	7–10	11–12
Regression intercept	− 7	6	–	8	27	–
Acro-TSQ change in EQ-VAS Unchanged group	− 8	5	–	6	6	–
Regression change for 10-point EQ-VAS change	5	6	–	6	8	–
Regression change for 15-point EQ-VAS change	7	8	–	9	13	–
Acro-TSQ change in EQ-VAS Much Improved group	12	13	–	10	28	–
Acro-TSQ change in EQ-VAS Much Worse group	− 14	− 19	–	− 11	− 4	–
MID estimate	10–12	9–11	–	8–10	Undeter-mined	–

*GI* gastrointestinal, *MID* minimally important difference

## Discussion

The first study found good internal consistency reliability, and convergent and known groups validity for the Acro-TSQ; however, test–retest reliability was below the established threshold for some scales, responsiveness could not be established because patients were relatively stable over time, and the MID could not be determined [15, 16]. There were also some ceiling effects identified in the first study for items within the Injection Site Reaction Interference and Emotions scales. After modifying some items, the EFA from the second study yielded 6 scales: Symptom Interference, Treatment Convenience, Injection Site Interference, GI Interference, Treatment Satisfaction, and Emotional Reaction. Scales identified from EFA had good to excellent reliability, and there was strong support for convergent validity and responsiveness. The MID was estimated for 3 scales: 10–12 points for Symptom Interference, 9–11 points for Treatment Convenience, and 8–10 points for GI Interference.

The Acro-TSQ was designed specifically for use with patients with acromegaly receiving injectable SSA to assess the impact of symptoms and treatment on their HRQoL. It is a brief, yet comprehensive measure that was developed according to current PRO standards. The results presented herein indicate that it is a valid, reliable, and responsive tool for use in this population, and the tool is available upon request for use in practice and clinical trials. The content of the Acro-TSQ is specific to symptom interference, treatment convenience, injection site interference, GI interference, treatment satisfaction and emotional Reaction. Therefore, all of the items except those pertaining to injection site interference would be relevant to individuals receiving any type of acromegaly treatment with any mode of administration. Further, the Acro-TSQ would be relevant for use in a clinical study, including a randomized or an observational study, or to assist with decision making within a clinical practice*.* The MIDs provide clinicians and researchers with guidelines for what constitutes clinically relevant change for 3 of the scale scores, while scales without guidelines for clinically relevant change can still be used to descriptively assess change. Strengths of this analysis include its development in a prospective large international study, the use of data that reflected the inclusion of a variety of collateral measures along with the use of both anchor- and distribution-based methods to estimate the MIDs.

The results should also be viewed in light of some limitations. The specific limitations of the first study (in terms of the high ceiling effects for several items, inability to examine responsiveness and a lack of suitable anchors to estimate a MID) were addressed in the second study. However, in the second study few patients experienced a worsening of symptoms, so sample sizes for the Unchanged and Worse groups for responsiveness were small overall. Finally, because of the study design it was not relevant for the Acro-TSQ version administered at Week 26 to include the Injection Site Interference scale and therefore longitudinal analyses, including responsiveness and estimating a MID, were not possible for this scale.

In addition to establishing the measurement properties of the Acro-TSQ, the results provide some additional insights regarding the assessment of disease severity for acromegaly patients. First, it is noteworthy that the GI Interference scale was correlated with AIS. This finding may suggest that the GI Interference scale reflects impacts of the underlying disease as well as treatment. Second, objective measures such as IGF-1 and AIS failed to significantly correlate to patient-reported severity of symptom interference. This suggests that certain aspects of symptomatology (e.g., the length of time symptoms persist, how they interfere with daily activities) not captured by these objective measures are important indicators of patients’ experiences with treatment. The Acro-TSQ is a patient-centered tool for assessing factors related to quality of life and treatment perception, and, when used in conjunction with clinical measures of disease activity like IGF-1, GH, and AIS, will provide a comprehensive picture of the impact of acromegaly and its treatment on patients’ lives.

Each of the Acro-TSQ scale scores produced correlations > 0.30 (in absolute value) with overall and/or scale scores of collateral measures. Three scales, Symptom Interference, GI Interference, and Treatment Satisfaction, showed correlations > 0.30 with AIS, WPAI:SHP, and EQ-5D-5L, while the other Acro-TSQ scales had correlations above 0.30 with aspects of one or two of the collateral measures. The fact that the Acro-TSQ scales correlate as expected with other widely accepted PROs used with this population demonstrates the validity of the Acro-TSQ. Additionally, because the Acro-TSQ addresses disease and treatment burden as well as patient satisfaction, it provides a more complete assessment than some other tools. For example, the Acromegaly Quality of Life Questionnaire (AcroQoL) [18, 30, 31] assesses physical signs and impacts to daily activities and social functioning, but does not address the effectiveness, symptom burden, or potential side effects of treatment. Similarly, the Acromegaly Disease Activity Tool (ACRODAT®) [32] does not address acromegaly treatment; it measures disease activity to support clinical decision-making. The SAGIT® assesses symptoms, comorbidities, and biochemical aspects of acromegaly [33], but is clinician-reported as opposed to patient-reported, and does not assess patient-reported measures of disease- or treatment-related HRQoL.

The Acro-TSQ is a novel PRO tool that can be used by clinicians to monitor patient outcomes associated with acromegaly treatment, and to track the extent to which symptoms are alleviated with efficacious treatments. It is valid and reliable, and suitable for use in clinical studies. Further research is needed to estimate the MID for Injection Site Interference, Emotional Reaction, and Treatment Satisfaction.

## Electronic supplementary material

Below is the link to the electronic supplementary material.

Supplementary file1 (DOCX 18 kb)
