# Supplementary material for: An evaluation of the Acromegaly Treatment Satisfaction Questionnaire (Acro-TSQ) in adult patients with acromegaly, including correlations with other patient-reported outcome measures: data from two large multicenter international studies
Source: Pituitary. 2020 Mar 27;23(4):347–58. doi: 10.1007/s11102-020-01038-y (PMC7316852; doi:10.1007/s11102-020-01038-y)
Supplement: Supplementary file 1 — Supplementary file1 (DOCX 18 kb) [file 11102_2020_1038_MOESM1_ESM.docx]

**Supplementary Material**

Descriptions of the Acromegaly Index of Severity (AIS), the Work Productivity and Activity Impairment Questionnaire Specific Health Problem V2.0 (WPAI:SHP), and the 5-level EQ-5D version (EQ-5D-5L

*AIS*

The AIS contains 5 items assessing the following symptoms: Headache, Swelling of extremities, Joint pain, Sweating, and Fatigue. Each symptom is graded by its highest severity during the last four weeks, from no symptoms (score 0), to mild symptoms (1), moderate symptoms (2) or severe symptoms (3). The AIS Overall Score is the sum of severity scores for each of the 5 acromegaly symptoms and has a possible range of 0 to 15, with 0 representing no symptoms and 15 representing severe symptoms. The AIS was completed at screening, baseline, and each scheduled visit during the run-in phase, including week 26

*WPAI:SHP*

The WPAI:SHP is a standardized, self-administered questionnaire applicable to a wide range of health conditions. It contains 6 questions regarding time missed from work, impairment of work, work productivity, and impact on regular activities. The WPAI:SHP yields four scores: Absenteeism (work time missed); Presenteeism (impairment at work / reduced on-the-job effectiveness); Work productivity loss (overall work impairment / absenteeism plus presenteeism); and Activity Impairment expressed as impairment percentages, with higher numbers indicating greater impairment and less productivity (i.e., worse outcomes). The WPAI:SHP was completed at screening, baseline, and week 26.

*EQ-5D-5L*

The EQ-5D-5L, developed by the EuroQoL, is also a generic self-administered questionnaire applicable to a wide range of health conditions containing five dimensions of health: Mobility, Ability to Self-care, Ability to Undertake Usual Activities, Pain and Discomfort, and Anxiety and Depression. The EQ- Visual Analogue Scale (VAS) records the patient’s self-rated health on a 20-cm vertical, visual analogue scale with endpoints labelled ‘the best health you can imagine’ (value of 100) and ‘the worst health you can imagine’ (value 0). The EQ-5D-5L was completed at screening, baseline, and week 26.

Additional detail regarding statistical methods

*Exploratory Factor Analysis*

For Exploratory Factor Analysis (EFA), the number of scales identified for the Acro-TSQ was determined on the basis of eigenvalues > 1.0, inspection of the SCREE plot, and rotated factor loadings. Items that had poor factor loadings (e.g., < 0.40 on any factor) or high cross-loadings on other factors (e.g., > 0.30 on any other factor) were dropped sequentially, and the analyses was repeated until a final factor model was reached. The second model included only variables that had factor loadings > 0.40 and cross-factor loadings < 0.30.

*Known Groups Validity*

For known groups validity in both studies, eta-squared was used to estimate the effect size. A minimum eta-squared of 0.06 (a “medium” effect size as defined by Cohen, 1988 [[1](#_ENREF_1)]) was required for evidence of known-groups validity.

*Responsiveness*

Regarding responsiveness, in the first study this was evaluated by calculating the standardized effect size (SES) [[1](#_ENREF_1)], the standardized response mean (SRM) [[2](#_ENREF_2)], and the responsiveness statistic (RS) [[3](#_ENREF_3)], using scores at baseline and month 3. These measures calculate how much the average score changes over time divided by some measure of overall variability: the SES uses the standard deviation at the initial time point, the SRM uses the standard deviation of the change score, and the RS uses the standard deviation of the change score among “stable” patients. Stable patients in the first study were defined as those individuals who rated themselves as unchanged on PGA at month 3.

For the second study, responsiveness of the Acro-TSQ scales was evaluated using the SES, SRM, and RS, as described for the first study, except that stable patients were defined as those individuals whose EQ-VAS score at Week 26 was within ± 5% of the EQ-VAS score at screening. Responsiveness coefficients were reported for the overall sample as well as separately by changes in AIS Overall Score and IGF-1 from screening to week 26. Changes in AIS Overall Score from screening to week 26 were calculated, and participants were grouped into three groups: Worse: week 26 > screening (N = 24); Unchanged: week 26 = screening (N = 19); and Improved: week 26 < screening (N = 34). Responsiveness coefficients were then calculated for each group on all Acro-TSQ scales except Injection Site Interference, which was not assessed at week 26. Changes in IGF-1 ULN values from screening to week 26 were calculated, and participants were classified into the following groups: Worse: > 5% increase in scores; Unchanged: ± 5% change in scores; and Improved: > 5% decrease in scores.

*MID*

For MID estimation in the first study, the primary anchors for determining the MID for the Acro-TSQ scales were the month 3 PGI-C and CGI-C. In the second study, the primary anchors were the EQ-VAS General Health Rating and IGF-1. The AIS Overall Score was used as an additional anchor for the Symptom Interference scale. Spearman correlation coefficients were calculated between changes in Acro-TSQ scales and changes in anchors from baseline to month 3 (first study) or screening to week 26 (second study). In the second study, the following EQ-VAS change groups were used: Much Worse: > 10 point decrease in EQ-VAS; Somewhat Worse: 1-10 point decrease in EQ-VAS; Unchanged: No change in EQ-VAS; Somewhat Improved: 1-10 increase in EQ-VAS; and Much Improved: > 10 point increase in EQ-VAS. Anchor-based analyses described below were only performed if the Spearman correlation was at least 0.30. Patients were categorized into groups of improved, stable, and worsened, based on their changes in anchors. Mean changes in Acro-TSQ scale scores from baseline to month 3 (first) or screening to week 26 (second) were reported separately by group.

In the second study, ordinary least squares regression analysis was conducted using changes in anchors from screening to week 26 and the anchor score at screening to predict changes in Acro-TSQ scale scores from screening to week 26. Screening anchor scores were centered so that a value of zero represented the average anchor score at screening.

Distribution-based methods, including the 1.0 standard error of measurement (SEM) [[4](#_ENREF_4)], SES, and the RS, were used as supportive information to establish thresholds for clinically meaningful change.

*References*

1. Cohen, J.: Statistical Power Analysis for the Behavioral Sciences, Second Edition. Lawrence Erlbaum, Hillsdale, New Jersey (1988)

2. Stucki, G., Liang, M.H., Fossel, A.H., Katz, J.N.: Relative responsiveness of condition-specific and generic health status measures in degenerative lumbar spinal stenosis. Journal of clinical epidemiology **48**(11), 1369-1378 (1995).

3. Guyatt, G.H., Bombardier, C., Tugwell, P.X.: Measuring disease-specific quality of life in clinical trials. CMAJ : Canadian Medical Association journal = journal de l'Association medicale canadienne **134**(8), 889-895 (1986).

4. Crosby, R.D., Kolotkin, R.L., Williams, G.R.: Defining clinically meaningful change in health-related quality of life. Journal of clinical epidemiology **56**(5), 395-407 (2003).
